# Clinical Characteristic Analysis of Seven Children With Bickerstaff Brainstem Encephalitis in China

**DOI:** 10.3389/fneur.2020.00557

**Published:** 2020-06-12

**Authors:** Yifeng Ding, Lifei Yu, Shuizhen Zhou, Linmei Zhang

**Affiliations:** Department of Neurology, Children's Hospital of Fudan University, Shanghai, China

**Keywords:** Bickerstaff brainstem encephalitis, pediatric, clinical characteristics, anti-GQ1b antibody, China

## Abstract

**Objective:** To summarize the clinical, electrophysiological, neuroimaging, and immunological characteristics of seven cases of Bickerstaff brainstem encephalitis (BBE) in China and to determine whether certain clinical features suggestive of BBE can facilitate diagnosis and treatment.

**Patients and Methods:** The clinical data of seven BBE patients treated at the Children's Hospital of Fudan University between 2016 and 2019 were retrospectively analyzed. The clinical and laboratory characteristics of the BBE patients were studied.

**Results:** The seven patients in this study included four females and three males, and the median age at diagnosis was 5.3 years (interquartile range: 3.0–7.1 years). All seven patients had an acute onset with a preinfection history. Seven cases of acute extraocular paralysis, ataxia, and an impaired level of consciousness, two cases of tendon hyperreflexia, one case of positive pathology, and five cases of cranial nerve involvement (the facial nerve and oculomotor nerve) were noted. Cerebrospinal fluid (CSF) examination of five patients showed albuminocytologic dissociation. Electromyography (EMG) was used to examine seven patients; the results were normal in four patients, showed axonal involvement in two patients, and showed demyelination in one patient. The head magnetic resonance imaging (MRI) results of all seven patients were normal. Electroencephalogram (EEG) background activity in the five monitored patients was slowed down. Seven patients underwent serum antibody testing, three of whom were positive for anti-GQ1b antibody, while one was positive for anti-GM1 antibody. Three patients received glucocorticoid combined with intravenous immunoglobulin (IVIG) therapy, and four received only IVIG therapy. One patient required a nasal catheter for oxygen during the disease course, and left upper limb muscle dysfunction (grade III muscle strength of the left upper limb) was observed during the 6-month follow-up. The other six patients had a good prognosis and no dysfunction.

**Conclusion:** Our study identified clinical, imaging, and treatment characteristics that may have prognostic value for pediatric BBE. The positive rate of head MRI was low, the positive rate of serum anti-GQ1b ganglioside antibody was low, and the therapeutic effect of IVIG therapy was good.

## Introduction

In 1951, Bickerstaff and Cloake first reported three patients with drowsiness, ophthalmoplegia, and ataxia (1) and suggested that these symptoms might be caused by midbrain injury. Six years later, Bickerstaff reported five similar cases (2) and named this disease Bickerstaff brainstem encephalitis (BBE). BBE is an immune-mediated nervous system disease characterized by the triad of ataxia, encephalopathy, and ophthalmoplegia and affects both the brainstem and the peripheral nervous system (PNS). Currently, BBE is grouped with Guillain-Barré syndrome (GBS) and Miller-Fisher syndrome (MFS) in the same spectrum of diseases (3–7). BBE may be underreported due to the lack of specific clinical criteria and biomarkers. Many large-scale studies in Japan have reported that the annual incidence of BBE is ~8/100,000 (5, 8). However, very few studies have investigated pediatric BBE; the incidence rate is unknown, and pediatric BBE reports are very rare in China (9). This study retrospectively analyzed the clinical data of seven pediatric BBE patients who were diagnosed at the Department of Neurology of the Children's Hospital of Fudan University from 2016 to 2019 and reviewed the literature to determine whether certain clinical features suggestive of BBE can inform its diagnosis and treatment.

## Patients and Methods

### Patients

We included seven patients with clinically diagnosed BBE in this study. The inclusion criteria were as follows: (1) acute ophthalmoplegia, ataxia, and an impaired level of consciousness. If no limb weakness or muscle strength ≥IV is noted, then BBE is indicated; if limb weakness is evident (muscle strength ≤ III in any segment of the four limbs), then an overlapping form of BBE/GBS is indicated (10–12); and (2) an age of onset younger than 16 years ( ≤ 16 years). The exclusion criteria were as follows: diseases that can cause similar clinical manifestations, such as combined brainstem vascular disease, multiple sclerosis, Lyme disease, Wernicke's encephalopathy, neuro-Behçet's disease, botulinum toxin poisoning, myasthenia gravis, brainstem tumor, pituitary apoplexy, vasculitis, lymphoma, acute disseminated encephalomyelitis, and Creutzfeldt-Jakob disease.

### Methods

In this study, we retrospectively analyzed seven patients diagnosed with BBE at the Children's Hospital of Fudan University between November 2016 and January 2019. All patients met the inclusion criteria. The phenotypic data were assessed, including the presence or absence of drowsiness, muscle weakness, pathological signs, cranial nerve involvement, and ataxia. Electromyography (EMG), nerve conduction velocity (NCV) head magnetic resonance imaging (MRI), and electroencephalogram (EEG) were performed on all BBE patients. In all patients, serum IgG antibodies of the gangliosides GQ1b, GT1b, GD1b, GD1a, GM1, GM2, and GM3 were screened by enzyme-linked immunosorbent assay (ELISA), and GQ1b antibody was detected in the cerebrospinal fluid (CSF) of five patients. The ethics committee of the Children's Hospital of Fudan University approved this study.

## Results

### General Characteristics of the Patients

From November 2016 to January 2019, we recruited seven BBE patients from the Department of Neurology, Children's Hospital of Fudan University, Shanghai, China, including four females and three males. The median age at the initial diagnosis was 5.3 years (interquartile range: 3.0-7.1 years, Table 1).

**Table 1:** Clinical characteristics of the seven patients.

**Patient**	**Gender**	**Age of onset (m)**	**Clinical classification**	**Preinfection history**	**Peak of disease severity (d)**	**Limb weakness**	**Muscle strength**	**Pyramidal signs**	**Ophthalmoplegia**	**Diplopia**	**Bulbar paralysis**	**Alerted level of consciousness**
1	M	85	BBE	MI	8	Yes	IV	No	Yes	Yes	Yes	Yes
2	F	44	BBE	URI	12	Yes	V	No	Yes	Yes	No	Yes
3	F	163	BBE	URI	7	Yes	IV	No	Yes	Yes	No	Yes
4	F	65	BBE/GBS	URI	5	Yes	II	Yes	Yes	Yes	No	Yes
5	M	44	BBE/GBS	MP	9	Yes	III	Yes	Yes	Yes	No	Yes
6	F	36	BBE/GBS	Pneumonia	8	Yes	I	Yes	Yes	No	No	Yes
7	M	10	BBE	Pneumonia	7	Yes	V	No	Yes	No	Yes	Yes

*BBE/GBS, the overlapping form of BBE/GBS; MI, Mycoplasma infection; URI, Upper respiratory tract infection; MP, Mycoplasmal pneumonia*.

### Clinical Manifestations

All seven patients had an acute onset with a history of precursor infection. Four patients had pneumonia (two with mycoplasma infection), and three had acute upper respiratory tract infection.

The peak of disease severity was reached in 5 to 12 days from symptom onset (average time: 8 days), and all seven BBE patients showed drowsiness or varying degrees of an impaired level of consciousness, with pediatric Glasgow Coma Scale (GCS) scores of 8–13, ophthalmoplegia, and ataxia. Five patients had concurrent limb weakness, and three patients had muscle strength < III. Five patients had diplopia, and five patients had facial paralysis. Two cases of bulbar paralysis and two cases of tendon hyperreflexia were noted. One patient had a positive Babinski sign (Table 1). Only one patient developed dyspnea, difficulty discharging airway secretions, and carbon dioxide retention and was transferred to the ICU on the fifth day of the disease. However, 7 days later, without invasive ventilation, she spontaneously improved and was returned to the neurology ward.

Lumbar puncture (LP) was performed in seven patients at 2–3 weeks in the disease course, and CSF albuminocytologic dissociation was identified in five cases. Four cases of BBE and three cases of the overlapping form of BBE/GBS were noted. Four of seven patients were positive for serum antibodies, three of whom were positive for anti-GQ1b antibody, while one was positive for GM1 antibody. Five patients with CSF ganglioside antibodies tested negative (Table 2).

**Table 2:** Laboratory characteristics of the seven patients.

**Patient**	**CSF leukocyte (×10^**6**^/L)**	**CSF albumen** **(mg/L)**	**CSF glucose (mmol/L)**	**Serum** **GQ1b**	**Serum** **GM1**	**CSF** **GQ1b**	**CSF** **GM1**	**MRI**	**EEG**	**EMG/NCS**
1	0	963.0	4.1	+	−	−	−	N	S	N
2	0	228.8	4.3	−	+	−	−	N	S	N
3	1	475.4	3.9	+	−	−	−	N	N	N
4	0	874.6	4.6	+	−	−	−	N	S	AI
5	10	798.0	4.7	−	−	−	−	N	N	D
6	5	1537.3	5.7	−	−	−	−	N	S	AI
7	0	256.3	4	−	−	−	−	N	S	N

*CSF, cerebrospinal fluid; +, positive; −, negative; N, normal; S, background activity slowing in the EEG; AI, axonal involvement (the amplitude of the distal compound muscle action potential decreased by more than 20%); D, demyelination (motor NCV was reduced by more than 20%, with prolonged distal motor latency or decreasing F-waves)*.

All patients with the overlapping BBE/GBS form had alterations in either EMG or NCV. The amplitude of the distal compound muscle action potential decreased by more than 20%. Axonal involvement was the main manifestation in two patients. Motor NCV was reduced by more than 20%, with prolonged distal motor latency or decreasing F-waves. Demyelination was the main manifestation. The EMG/NCV results of the four patients with classic BBE were normal. The seven cranial and two spine plain MRI results were normal. EEG revealed abnormal background activity in five of the seven patients (Table 2).

All patients were treated with intravenous immunoglobulin (IVIG) (400 mg/kg/d) for 5 days. Two patients received a high dose of glucocorticoid (methylprednisolone, 20 mg/kg/d) for 3 days followed by oral prednisone treatment (2 mg/kg/d) for 2 weeks, and the dose was then gradually reduced until withdrawal after 1 month. Seven patients were closely followed for 10–34 months (average 19 months). Six patients recovered completely, three of whom fully recovered at 1–2 months, while three fully recovered at 5–12 months following symptom occurrence. The mean overall time for clinical recovery was 5 ± 4.3 months. The ataxia recovery time was 1–2 weeks, and the respiratory insufficiency recovery time was 2–6 weeks. Decreased muscle strength persisted for longer periods ranging from 1 to 21 weeks. Patient No. 4 was followed up to 21 months: ataxia and respiratory insufficiency had recovered completely, but the left upper limb muscle strength had not returned to normal (muscle strength level III). None of the patients relapsed during the follow-up period (Table 3).

**Table 3:** Treatment and prognosis of the seven patients.

**Patient**	**IVIG**	**Steroids**	**Follow-up time (months)**	**Symptoms completely disappeared (Months)**
1	Yes	No	34	2
2	Yes	No	21	5
3	Yes	Yes	20	2
4	Yes	No	21	–
5	Yes	Yes	18	12
6	Yes	No	10	8
7	Yes	No	9	1

–, *not fully recovered*.

## Discussion

Pediatric BBE is a rare and frequently misdiagnosed disease. Both BBE and MFS have common infectious triggers (e.g., mycoplasma pneumonia), similar clinical manifestations and changes in CSF (such as albuminocytologic dissociation), and the same autoimmune antibodies (GQ1b) and cannot be differentiated by etiology or anatomy (5, 13). In 2009, Yuki proposed that these two diseases may have the same pathological mechanism, which is often called “Fisher-Bickerstaff syndrome” (14). Later, in 2013, Shahrizaila and Yuki proposed the concept of anti-GQ1b antibody syndrome (15). The difference between BBE and MFS might be that GQ1b is expressed in different parts in the two diseases. The presence of anti-GQ1b antibody may indicate that the complex symptoms of the patients included in our study were different manifestations of the same spectrum of diseases affecting the central nervous system (CNS) and the PNS to varying degrees (13, 15). BBE mainly affects the CNS, while MFS mainly affects the PNS. Encephalopathy is the most important distinguishing characteristic between BBE and MFS. We also agree with Michev and Coll that if areflexia and limb weakness exist simultaneously, the condition should be defined as the overlapping form of BBE/GBS; that is, the CNS and PNS are involved simultaneously, which is different from classical BBE (16). A study has confirmed (17) that human brain microvascular endothelial cells (BMECs) in BBE patients can destroy the blood-brain barrier (BBB) through the autocrine secretion of matrix metalloproteinase-9 (MMP-9), thereby causing CNS-related symptoms, while the serum of MFS patients had no destructive effect on the BBB. Studies suggest that although children are more likely to have abnormalities in LP, MRI, EMG/NCV, and EEG findings, the clinical profiles are similar to those of adults (6, 8, 15).

In 1992, Chiba et al. (18) found anti-GQ1b antibody in patients with Fisher syndrome (FS), which prompted research into the mechanism of FS and was followed by reports of autoantibodies in BBE patients (19); therefore, the concept of 'Fisher-Bickerstaff syndrome' has been developed (14). Globally, the positive rate of GQ1b antibodies in pediatric BBE patients is 43–68% (7, 20). The positive rate of antibodies is related to the disease course (16, 21). In our study, the GQ1b antibody positive rate was 42.86%, which is essentially consistent with international levels (20); however, the sample size must be expanded to accurately evaluate the positive rate of GQ1b antibodies in Chinese pediatric BBE. Although we tested all patients for GQ1b antibodies within 4 weeks of the disease course, we could not reach the 68% positive rate described in Santoro JD (7).

The prognosis of pediatric BBE patients is mostly good. Considering that IVIG can shorten the recovery time of pediatric GBS patients (22, 23), a study has shown (7) that IVIG or plasmapheresis might reduce the overall duration of symptoms and is gradually becoming accepted as a first-line treatment for BBE (24–26). Of course, symptomatic supportive care during the disease course is also essential (27). Just as glucocorticoids are not effective for treating GBS, glucocorticoids may also not be effective for BBE. In this study, two patients received glucocorticoid therapy at the same time, but a study with a large sample size is needed to determine the effectiveness of glucocorticoid treatment for BBE. Additionally, one patient with an overlapping form of BBE/GBS had muscle strength of 0-I at the most severe point of the disease, and muscle weakness in one limb persisted at the 21st month of follow-up (the muscle strength of the left upper limb was grade III). These results suggest that lower muscle strength in patients with the overlapping form of BBE/GBS corresponds to a slower recovery.

This study has some limitations. Pediatric BBE is a rare disease, and this study was not a multicenter study with a large sample size; therefore, future studies with large sample sizes are needed to support our conclusions. In this study, no enhanced head MRI was performed; therefore, small lesions in the brainstem region may not have been detected. Although the longest follow-up in this study was 34 months (mean 19 months), a longer follow-up is needed to confirm the relatively good prognosis of BBE and the absence of recurrence.

## Conclusion

Children with BBE usually show an acute onset and often have presymptomatic infections from viruses, bacteria, or mycoplasmas. BBE often has a monophasic course. Pediatric patients with the overlapping form of BBE/GBS are commonly encountered. The rate of positive serum ganglioside antibodies is not high, and IVIG is an effective treatment. Ataxia recovers the fastest, and respiratory function recovery is good after appropriate respiratory support is provided. The prognosis of simple BBE may be better than that of the overlapping form of BBE/GBS. Lower muscle strength in patients with the overlapping form corresponds to a slower recovery. The diagnosis of pediatric BBE is mainly based on clinical characteristics. A specific immunological examination is helpful for confirming the diagnosis. In addition, comprehensive neuroimaging and neurophysiological examination can help determine the clinical classification and prognosis.

## Data Availability Statement

All datasets generated for this study are included in the article/supplementary material.

## Ethics Statement

The studies involving human participants were reviewed and approved by the Ethics Committee of the Fudan University Children's Hospital. Written informed consent from the participants' legal guardian/next of kin was not required to participate in this study in accordance with the national legislation and the institutional requirements.

## Author Contributions

YD and LY designed the research, wrote the manuscript, and approved the submitted version. YD, SZ, and LZ collected and analyzed the data.

## Conflict of Interest

The authors declare that the research was conducted in the absence of any commercial or financial relationships that could be construed as a potential conflict of interest.

## Abbreviations

BBE: Bickerstaff brainstem encephalitis
CSF: cerebrospinal fluid
EMG: electromyography
NCV: nerve conduction velocity
MRI: magnetic resonance imaging
EEG: electroencephalogram
IVIG: immunoglobulin
GBS: Guillain-Barré, syndrome
MFS: Miller-Fisher syndrome
ELISA: enzyme-linked immunosorbent assay
PNS: peripheral nervous system
CNS: central nervous system
BMECs: brain microvascular endothelial cells
BBB: blood-brain barrier
MMP-9: matrix metalloproteinase-9
BBE/GBS: the overlapping form of BBE/GBS
MI: mycoplasma infection
URI: upper respiratory tract infection
MP: mycoplasma pneumonia
CSF: cerebrospinal fluid
LP: lumbar puncture.
